# Etoposide and cisplatin in combination with anlotinib for lung NUT carcinoma: a case report

**DOI:** 10.3389/fonc.2025.1632133

**Published:** 2025-09-17

**Authors:** Yuxing Sun, Jiangyu Bian, Linfeng Wang, Tong Zhang

**Affiliations:** ^1^ Department of Oncology, Xiyuan Hospital of China Academy of Chinese Medical Sciences, Beijing, China; ^2^ China Three Gorges University, Yichang, Hubei, China

**Keywords:** lung NUT carcinoma, NUT midline carcinoma, chemotherapy, anlotinib, targeted therapy, case report

## Abstract

Lung NUT carcinoma is a rare malignant tumor, which is highly aggressive, poorly differentiated, and difficult to recognize at an early stage, and is associated with very rare reports and extremely poor prognosis, with some reports showing a mOS of only 2.2 months. In this paper, we report the treatment of a rare case of primary lung NUT cancer. After surgery, chemotherapy and targeted therapy, the patient’s progression-free survival is now more than 4 months, which provides a feasible treatment option for lung NUT cancer.

## 1 Introduction

Pulmonary NUT carcinoma belongs to a type of Nuclear protein of the testis carcinoma(NC).NC also known as NUT midline carcinoma (NMC), is a rare and highly aggressive malignant tumor that can occur anywhere in the body but most tumors are located in the midline anatomy or mediastinum and are characterized by chromosomal rearrangements (1). Due to its rarity, no studies have been conducted to clarify the specific incidence values and the pattern of disease incidence distribution, and only partial evidence-based evidence suggests that the onset of the disease involves patients of all age groups and is more common in younger patients (2–4). Cases of pulmonary NUT carcinoma are reported to be rare and have a poor prognosis, with a median overall survival (OS) of only 2.2 months in a real-world retrospective study (5). In this paper, we report a case of pulmonary NUT carcinoma with a long survival after comprehensive treatment including surgery and chemotherapy combined with antivascular targeted therapy, which provides a reference for the clinical management of pulmonary NUT carcinoma.

## 2 Case presentation

**Figure 1:**
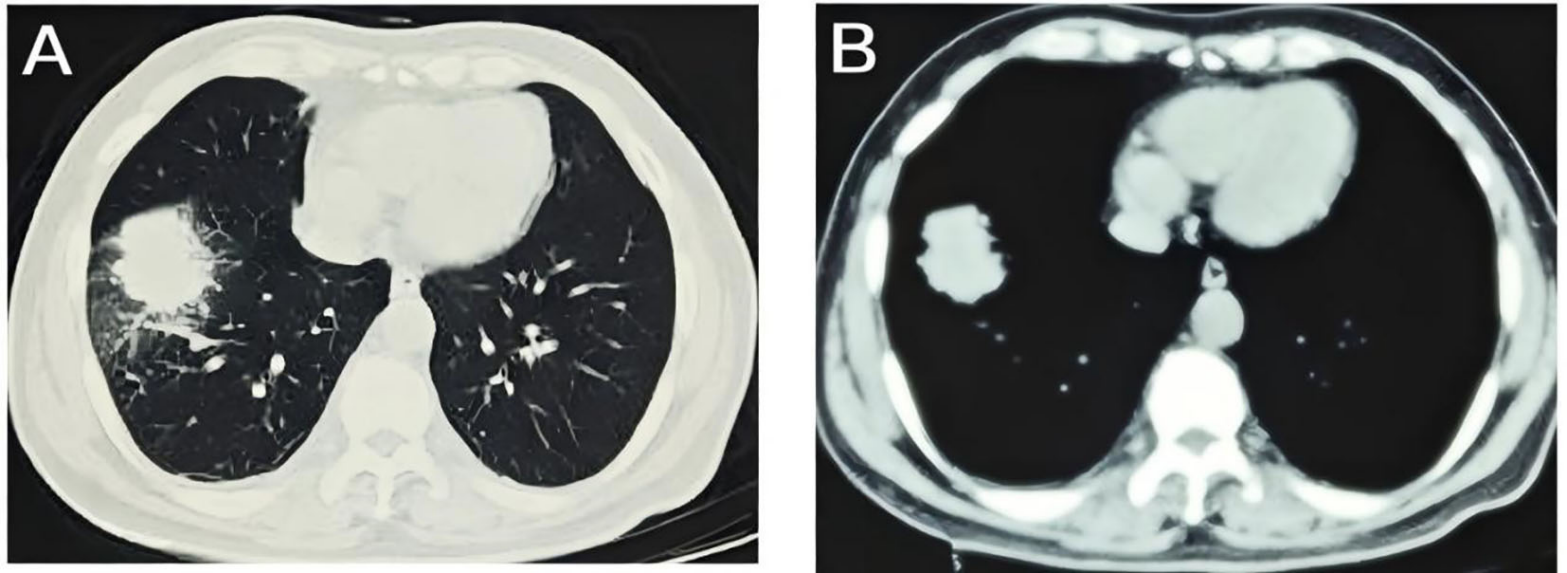
Baseline imaging characteristics: CT imaging on September 18, 2024, established the initial diagnosis of pulmonary NUT carcinoma; **(A)** lung window; **(B)** mediastinal window.

**Figure 2:**
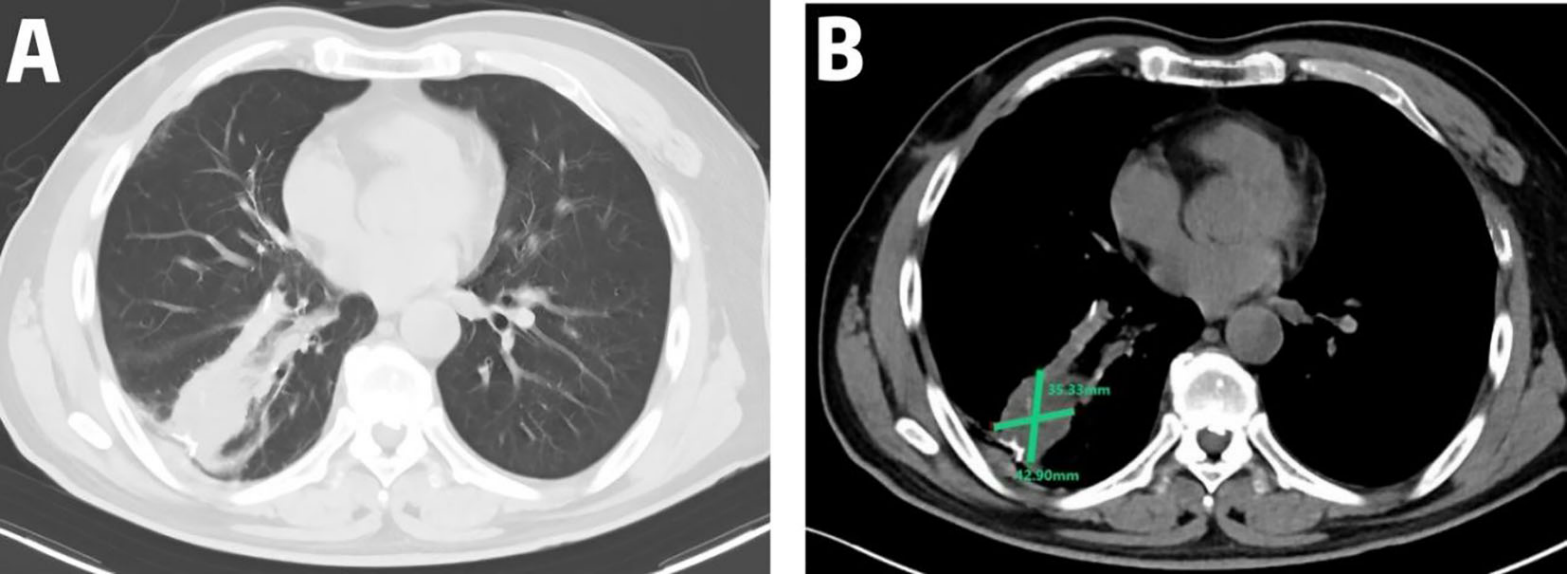
Disease progression on targeted therapy: CT imaging on January 15, 2025, demonstrated a peripheral soft tissue opacity in the right lower lobe (42.90 × 35.33 mm) showing interval enlargement compared to pre-treatment baseline; **(A)** lung window; **(B)** mediastinal window.

**Figure 3:**
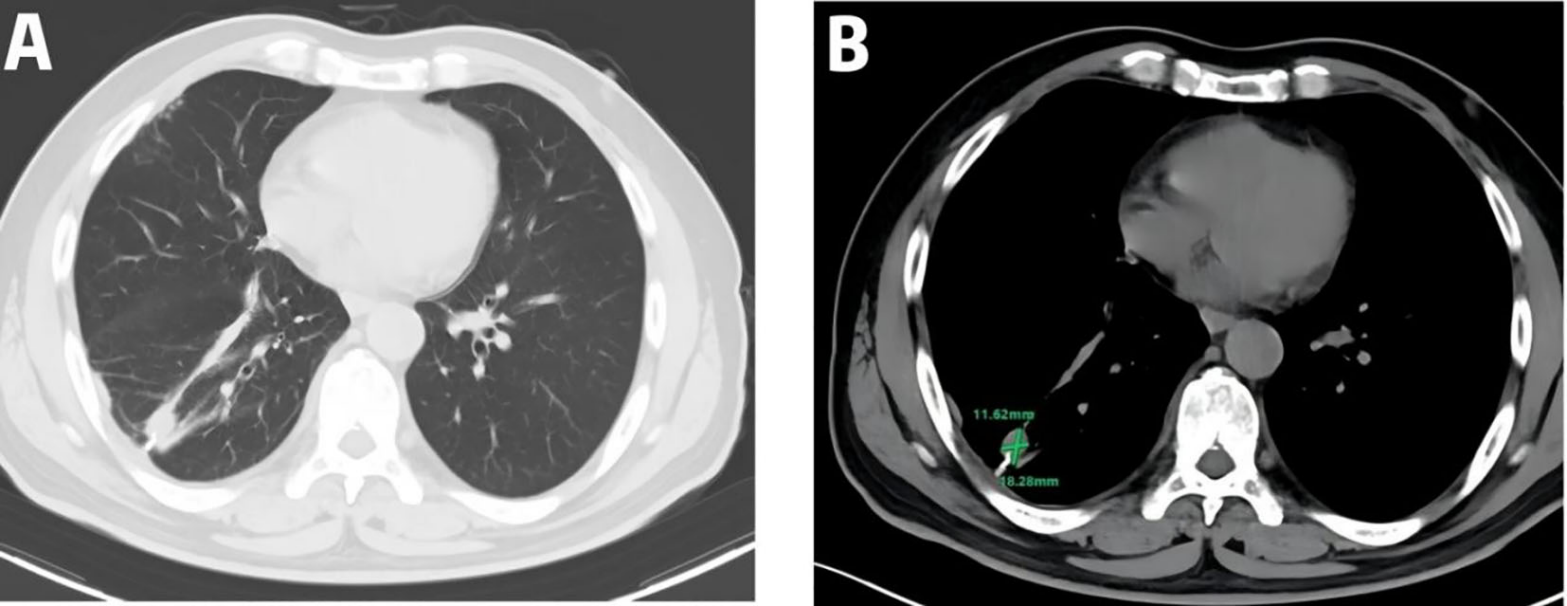
Therapeutic response to EP plus anlotinib: Post-treatment CT on April 7, 2025 (after 4 cycles) showed a 25% reduction in the target lesion (18.28 × 11.62 mm) compared to the January 15, 2025 assessment with no new metastatic lesions observed; **(A)** lung window; **(B)** mediastinal window.

A 54-year-old male presented to the local hospital in September 2024 with a cough. No family history of genetic predisposition. Chest CT (**Figure 1**) showed a right lower lobe occupancy of about 47*41 mm with multiple tiny nodules in both lungs. PET/CT showed a right lower lobe hypermetabolic occupancy of about 47*41 mm with SUVmax: 11.1 and right hilar hypermetabolic lymph node with SUVmax: 8.3.The patient underwent video-assisted thoracic surgery for partial lower lobectomy of the right lung along with excision of a diaphragmatic mass at Dezhou Hospital of Shandong University Qilu Hospital on September 27, 2024.Postoperative pathology: NUT carcinoma (right lung lower lobe mass). Immunohistochemistry: NUT (1+), P40 (2+), INSM1 (-), CK5/6 foci (+), CgA (-), Syn (-), CD56 (-), ALK (-), HER2 (0), Ki67 (70%+). Genetic testing (PAN116) showed no driver mutations and Microsatellite Stability (MSS). Lung NUT carcinoma was considered in combination with immunohistochemistry. No postoperative treatment was performed and chest CT was repeated in November 2024 and Progressive Disease (PD) was evaluated according to Response Evaluation Criteria in Solid Tumors 1.1 (RECIST 1.1) efficacy. Genetic testing was performed to see APC, ATM, BRCA1, BRCA2, CSFIR, GNA11, KIT, MLH1, EGFR, and ROS1 mutations, TMB 2mut/Mb, and MSS. One cycle of treatment was performed in December 2024 at outside hospital, with the following specific regimen: anlotinib (8mg qd), afatinib (30mg qd), and olaparib (150mg bid). Review CT in January 2025 suggested (**Figure 2**) postoperative changes in the lower lobe of the right lung, peripheral strips of soft-tissue shadows, which increased in size compared with the range of 2024-11, fullness of the right lung hilar, no significant changes were seen, thickening and adhesion of the right pleura, and a small amount of effusion in the right thoracic cavity. Efficacy evaluation of PD. Four cycles of etoposide (1.8g d1-3), cisplatin (60mg d1-2), and anlotinib (8mg po d1-14,q21d) were performed on 2025-1-16, 2-6, 2-26, and 3-18.A repeat CT (**Figure 3**) on 2025-4–7 suggested postoperative changes in the right lower lobe of the lungs, with striated solid changes in the operative area, and a slightly enlarged lymph node in the right hilar region, with a large one 8mm in short diameter. Right pleural thickening and adhesion, right pleural cavity little effusion roughly the same as before, compare 2025-3–18 did not see obvious changes. The current tumor measures 18.28*11.62 mm, with a 25% reduction in the sum of the longest diameters of target lesions compared to the January 15, 2025 baseline without new lesions, meeting the criteria for partial response (PR) according to RECIST 1.1. At present, the patient is still in the second-line chemotherapy combined with targeted therapy, the current PFS has been more than 3 months, the patient’s treatment was well tolerated, and the symptoms of cough and fatigue were once significantly improved compared with the pre-treatment period. The patient’s treatment history is shown in **Table 1**.

**Table 1:** The patient’s treatment history.

Timepoint	Intervention	PFS months	Assessment	Key outcomes
2024-09-27	Video-assisted thoracic surgery for partial lower lobectomy of the right lung along with excision of a diaphragmatic	3	Postoperative pathology and IHC	Pathologically confirmed NUT carcinoma
2024-12	Anlotinib (8 mg qd) Afatinib (30 mg qd) Olaparib (150 mg bid)	1	RECIST 1.1, PET/CT, CT	Target lesion: Metabolic hyperintensity 31*26*44mm, SUVmax6.9
2025-01-16	EP + Anlotinib (Cycle 1)	4+	RECIST 1.1, CT	Target lesions stable, no new metastases
2025-03-18	EP + Anlotinib (Cycle 4)		RECIST 1.1, CT	25% reduction in sum of longest diameters of target lesions vs. January 15, 2025 baseline; no new lesions; partial response (PR) per RECIST 1.1

## 3 Discussion

Diagnosing NUT midline carcinoma (NMC) poses significant challenges due to non-specific clinical manifestations and histopathological features lacking pathognomonic morphology. Current diagnostic approaches rely on immunohistochemical (IHC) staining demonstrating nuclear NUT overexpression or molecular confirmation of NUTM1 rearrangement through fluorescence *in situ* hybridization, next-generation sequencing, or reverse transcription polymerase chain reaction (6). In this case, the diagnosis of primary pulmonary NUT carcinoma was established based on poorly differentiated carcinoma identified via core needle biopsy, combined with NUT overexpression on IHC, and further supported by clinical presentation and imaging findings.

Pulmonary NUT carcinoma exhibits non-specific clinical manifestations unrelated to smoking, with cough being the most frequent symptom (7). The poorly differentiated or undifferentiated nature of the tumor complicates histomorphological identification and necessitates broad differential diagnoses. Focal squamous differentiation is frequently observed in pulmonary NUT carcinoma histology, where immunomarkers P63 and P40 may show positivity—features overlapping with pulmonary squamous cell carcinoma (SCC). Consequently, primary pulmonary NUT carcinoma was historically classified as an SCC subtype (8) but was reclassified under ‘other epithelial tumors’ in the 2015 and 2021 WHO classifications of thoracic tumors.

For thoracic NUT carcinoma, CT imaging delineates primary lesion size, location, lymph node involvement, and extent of local invasion. CT typically reveals a solitary lobulated mass without site predilection, characterized by large dimensions, relatively well-defined margins, and heterogeneous mild enhancement. Distant metastases to bone or lymph nodes are common (9, 10). In this case, initial CT demonstrated a 47×41 mm right lower lobe mass with bilateral pulmonary micronodules. PET/CT confirmed absence of distant metastasis.

The pathologic features, imaging manifestations, and standard treatment options for NUT cancers originating in the lung are currently unknown. Synchronized radiotherapy or sequential radiotherapy plays an important role, followed by surgery combined with neoadjuvant therapy or adjuvant radiotherapy. Surgery alone or chemotherapy alone also prolongs patient survival to some extent. The clinical efficacy of immunotherapy is unknown, and it is mainly used as a backline treatment. Chemotherapy regimens usually use platinum-containing dual agents (mainly TP). Anthracyclines, cyclophosphamide, isocyclophosphamide and other chemotherapeutic agents are also used (11). The EP regimen was selected for this case based primarily on germ cell tumor (GCT)-like characteristics: Involvement of testis-specific protein genes suggests potential germ cell tumor origin, and EP serves as the chemotherapeutic backbone for GCTs (12). Additionally, strong co-expression of P40 and CK5/6 indicates squamous differentiation. Studies have demonstrated EP application in SCC carcinomas under specific clinical circumstances (13). A Ki-67 index of 70% reflects aggressive tumor biology. Etoposide preferentially targets rapidly proliferating cells by stabilizing topoisomerase II-DNA complexes during S/G2 phase, while high Ki-67 tumors exhibit heightened mitotic activity, potentially indicating increased susceptibility to etoposide (14).

Angiogenesis plays an important role in tumor growth, and recent studies have shown that blocking angiogenesis has become a successful alternative strategy in cancer treatment (15). Anlotinib is a novel oral multi-target tyrosine kinase inhibitor that targets receptors involved in tumor proliferation, angiogenesis, and microenvironment modulation. It potently inhibits vascular endothelial growth factor receptor 2/3, fibroblast growth factor receptor 1-4, platelet-derived growth factor receptor α/β, stem cell factor receptor, and rearranged during transfection (16). This multi-kinase blockade confers broad antitumor activity against angiogenesis and growth in diverse solid tumors through three core mechanisms:Anti-angiogenesis: Suppresses VEGF-mediated endothelial cell proliferation, migration, and tube formation, significantly reducing tumor microvessel density. Direct antiproliferative action: Inhibits FGFR-dependent signaling pathways, induces G1/S cell cycle arrest, and triggers caspase-3-dependent apoptosis. Microenvironment modulation: Blocks PDGFR-β phosphorylation and downstream ERK signaling, inhibiting cancer-associated fibroblast activation and reducing interstitial fluid pressure (16). Anlotinib is currently approved as a third-line treatment for refractory advanced NSCLC, and recent studies have demonstrated that anrotinib increases the sensitivity of pneumoblastoma to *in vivo* chemotherapy (17). Therefore, we hypothesized that anlotinib may play an active role in overcoming chemotherapy resistance, and from imaging observations, chemotherapy combined with anlotinib as a second-line treatment for patients achieved PR, but the efficacy of this regimen remains to be further validated due to insufficient sample size.

## 4 Conclusion

Our first use of EP in combination with anlotinib significantly prolonged progression-free survival and significantly improved clinical symptoms in patients with lung NUT cancer, providing a therapeutic option for the treatment of lung NUT cancer.

## Data Availability

The original contributions presented in the study are included in the article/supplementary material. Further inquiries can be directed to the corresponding author.
